# Controlled human exposure to diesel exhaust: a method for understanding health effects of traffic-related air pollution

**DOI:** 10.1186/s12989-022-00454-1

**Published:** 2022-02-25

**Authors:** Erin Long, Carley Schwartz, Christopher Carlsten

**Affiliations:** 1 Faculty of Medicine, University of British Columbia, 317 – 2194 Health Sciences Mall, Vancouver, BC V6T 1Z3 Canada; 2 Department of Medicine, Division of Respiratory Medicine, University of British Columbia, 2775 Laurel Street 7th Floor, Vancouver, BC V5Z 1M9 Canada

**Keywords:** Controlled human exposure, Diesel exhaust, Particulate matter, Air pollution, Humans

## Abstract

Diesel exhaust (DE) is a major component of air pollution in urban centers. Controlled human exposure (CHE) experiments are commonly used to investigate the acute effects of DE inhalation specifically and also as a paradigm for investigating responses to traffic-related air pollution (TRAP) more generally. Given the critical role this model plays in our understanding of TRAP’s health effects mechanistically and in support of associated policy and regulation, we review the methodology of CHE to DE (CHE–DE) in detail to distill critical elements so that the results of these studies can be understood in context. From 104 eligible publications, we identified 79 CHE–DE studies and extracted information on DE generation, exposure session characteristics, pollutant and particulate composition of exposures, and participant demographics. Virtually all studies had a crossover design, and most studies involved a single DE exposure per participant. Exposure sessions were typically 1 or 2 h in duration, with participants alternating between exercise and rest. Most CHE–DE targeted a PM concentration of 300 μg/m^3^. There was a wide range in commonly measured co-pollutants including nitrogen oxides, carbon monoxide, and total organic compounds. Reporting of detailed parameters of aerosol composition, including particle diameter, was inconsistent between studies, and older studies from a given lab were often cited *in lieu* of repeating measurements for new experiments. There was a male predominance in participants, and over half of studies involved healthy participants only. Other populations studied include those with asthma, atopy, or metabolic syndrome. Standardization in reporting exposure conditions, potentially using current versions of engines with modern emissions control technology, will allow for more valid comparisons between studies of CHE–DE, while recognizing that diesel engines in much of the world remain old and heterogeneous. Inclusion of female participants as well as populations more susceptible to TRAP will broaden the applicability of results from CHE–DE studies.

**Supplementary Information:**

The online version contains supplementary material available at 10.1186/s12989-022-00454-1.

## Background

Exposure to air pollution is an important global health issue, and has recently been estimated to cause approximately 7 million deaths worldwide [1]. Traffic related air pollution (TRAP) is the largest contributor to air pollution in most urban centers and is responsible for 20–30% of global pollutant emissions [2]. TRAP exposure has been associated with various health outcomes including those associated with airways disease [3–6], cardiovascular disorders [7, 8] and a range of disturbances within other organs systems. Controlled human exposures (CHE) are a study design that is commonly used to investigate the acute effects of air pollution. The goal of CHE studies is to safely expose participants to a known amount of pollutant in a controlled environment to assess specific reversible health-related endpoints resulting from human exposure, without inducing overt clinical events. Air pollution exposures frequently employed in CHEs include diesel exhaust (DE) [9], concentrated ambient particles (CAP) [10], and wood smoke [11]. These types of studies are used, in conjunction with epidemiological, in vitro, and animal model studies, to provide biological plausibility and mechanistic insight and thus contribute to a more complete picture of pollution-related health outcomes. These studies are also used to test interventions that are thought to be of potential benefit, as evidence of effectiveness from such controlled studies may substantially increase confidence in the value of such interventions.

Advantages of CHE studies include the ability to control exposure duration, concentration, and other exposure-related factors. These types of CHE studies also typically benefit from a crossover experimental design that effectively eliminates risk of confounding by personal variables that pose inferential threats in other, most commonly observational, study designs. Another strength of CHE studies is the ability to select certain populations for investigation, allowing for more flexibility in the hypotheses that can be tested. Finally, CHE studies provide the capacity to assess a large range and number of clinically and biologically relevant endpoints efficiently within a single study [9]. The contribution of CHE studies has provided invaluable insight to our current understanding on the health impacts of air pollution, and, in turn has significantly buttressed regulatory imperatives in the face of challenges in the legal setting and otherwise.

DE from motor vehicles is a considerable contributor to TRAP given the extensive use of diesel engines in trucks, trains, boats buses, vans, and in some parts of the world, passenger cars. The main constituents of DE are nitrogen oxides (NO_x_), particulate matter (PM), carbon monoxide (CO), and a range of hydrocarbons (HC) all of which have be shown to threaten human health [12]. Emissions from diesel engines are the greatest contributor to NO_x_ derived from transportation and are also a significant source of PM [12]. As such, DE is a frequently used paradigm for TRAP exposure, particularly those acute, in studies of CHE to DE (CHE–DE). A complete and detailed characterization of the diesel exhaust particles (DEP) present in a CHE is essential to understanding the related health impacts. As such, CHE–DE typically report concentration of PM (which, though modest, for many studies appears enough to demonstrate acute effects of DE exposure [13–16]) and various fractions therein, particle size distribution, particle number, elemental and organic carbon (EC and OC respectively). Other exposure characteristics commonly reported include nitrogen monoxide (NO), nitrogen dioxide (NO_2_), NO_x_, CO, total volatile organic compounds (TVOC), temperature, and humidity. Engine load, fuel sulfur content, and newer engine technologies have been shown to affect DE composition with respect to both PM and gaseous emissions [17]. Variations in engine load have been shown to mediate differential effects on immune, cardiac, and pulmonary function in mice [18]. As such, other factors that affect the composition of diesel engine emissions likely influence health effects as well, underlining the importance of accuracy in reporting exposure characteristics in connection to measured health outcomes.

The purpose of this review is to provide a detailed documentation of experimental design, exposure conditions, and participant demographics for CHE–DE studies to date, for the purpose of understanding the results of those studies and caveats therein, along with implications for translation of associated results, and for then optimizing future experimental design. Earlier reviews of CHE–DE publications have discussed the findings derived from this body of literature with respect to the main health impacts of DE [9], or focused on a particular subset of CHE–DE experiments [19, 20]. This publication is the first to comprehensively review the methodologies of all CHE–DE studies published to date.

## Methods

### Literature search

A search of PubMed and Web of Science databases was conducted to identify English language CHE studies involving DE. All queries included the keyword “diesel exhaust” in combination with “exposure”, “controlled human exposure”, or “human exposure” (eg. “diesel exhaust” AND “exposure”). To be eligible, the publication had to expose participants to a controlled quantity of diesel exhaust via inhalation. Articles up until December 2020 were included. Letters, abstracts, and academic theses were excluded as they were subject to less rigorous peer review and/or provided less fulsome data for scrutiny. A search of the citations for each publication as well as the Clinicaltrials.gov registration page for publications that reported a clinical trial number was also conducted for eligible studies. This resulted in a total of 79 CHE–DE studies, with results reported across 104 eligible publications that were identified and reviewed. Publications reporting results from the same CHE–DE study were clustered within our data distillation.

### Data extraction

From these studies the following study details were extracted: method of diesel exhaust generation, key design elements including exposure arms, specific characteristics of DE and control exposures, and participant demographics. The specific parameters extracted are listed in Table 1.

**Table 1:** Study elements and specific parameters extracted from each study

Study element	Parameters extracted
Diesel exhaust generation	Engine, engine emission standards tier, fuel, load
Key study design elements	Study type, duration of exposure, number and type of exposures, activity during exposure, washout period, concurrent exposures, temperature, humidity
DE exposure composition	PM concentration, NO, NO_2_, NO_x_, CO, TVOC, formaldehyde, VOCs, HC compounds, EC and OC content, particle size, particle count
FA exposure composition	PM concentration, NO, NO_2_, NO_x_, CO, TVOC, formaldehyde, VOCs, HC compounds, EC and OC content, particle size, particle count
Participant characteristics	Inclusion criteria, exclusion criteria, sex, age, sample size
Other	Clinicaltrials.gov identifier

*CO* carbon monoxide, *DE* diesel exhaust, *EC* elemental carbon, *FA* filtered air, *HC* hydrocarbons, *NO* nitrogen monoxide, *NO*_*2*_ nitrogen dioxide, *NO*_*x*_ nitrogen oxides, *OC* organic carbon, *PM* particulate matter, *TVOC* total volatile organic compounds, *VOC* volatile organic compounds

Note that various studies reviewed had missing data for one or more of the components described above. If the study referenced an exposure system used previously under similar study design parameters, data from the most recent publication was extracted for this review. Missing data for some studies was obtained through correspondence with the study teams.

The results with respect to health outcomes of these 104 publications are reviewed in a separate companion paper [21].

## Results

### Method of diesel exhaust generation

A summary of all reported methods of diesel exhaust generation from reviewed studies are found in the supplemental material (see Additional file 1). A total of 18 DE generation methods were used across the 79 studies. Of these methods, 2 complied with Environmental Protection Agency (EPA) Tier 3, 1 with EPA Tier 1, 1 with European Union (EU) Stage 2, and 14 were uncategorized by emission standards. Yanmar, Volvo, Cummins were the most common brands of generators. Most fuel used was low or ultra-low sulfur diesel. Many studies used idling engines (31) though some opted to use stable or cycling loads to simulate conditions of real use. Two studies exposed participants to both DE produced under idling conditions and to DE produced under urban driving conditions [22, 23].

### Study design

A summary of reported CHE study design characteristics is shown in Table 2. With the exception of 4 studies (1 with single arm [108], 1 with single sequence [57], and 2 with parallel group design [54, 61]), all reviewed studies had a randomized crossover design, in which each participant underwent each exposure arm (acting as his/her own control). The vast majority of studies (75) included at least one filtered air (FA) or ambient air exposure as control. 46 studies included a single DE exposure arm, 25 had two DE exposure arms, and the remaining 8 studies had between 3 and 5 DE exposure arms. 3 studies had two different cohorts that underwent either one DE arm or two DE arms [66–68] and 2 studies had two or more different cohorts that underwent, per study protocol, either one DE arm or no DE arms [54, 61]. In this review, these different cohorts were considered separately and are represented as separate arms in all figures (e.g. if for the same study one cohort underwent one DE arm and the other cohort two DE arms, then three DE arms were considered for analysis in this review). Exposure sessions were usually 60 or 120 min in length, with washout periods of between 1 and 4 weeks between each exposure session for a given participant. 24 studies involved co-exposures to both DE and additional agents such as allergen, ozone (O_3_), antioxidant, and noise (Table 2).

**Table 2:** Summary of study design characteristics

Publication^a^	Study type	Exposure arms^b^	Length of exposure session (minutes)	Activity during exposure	Period between exposure sessions	Concurrent or additional exposures	Research group
Koch et al. [24]	Crossover	FA + placebo DE 300 + placebo FA + salbutamol DE 300 + salbutamol	90	60 min rest, followed by 30 min cycling at 50% peak power	1–3 weeks	Inhaled salbutamol	Air Pollution Exposure Laboratory, Vancouver, Canada
Li et al. [25]^c^	Crossover	DE 300 + allergen FA + saline FA + allergen	120	n/a	At least 4 weeks	Allergen	
Ryu et al. [15] Wooding et al. [26]		DE 300 + allergen PDDE + allergen FA + allergen FA + saline			4 weeks		
Rabinovitch et al. [27]	Crossover	DE 300 FA	120	n/a	At least 2 weeks	None	
Wooding et al. [28]	Crossover	DE 300 FA	120	Cycling on stationary bike twice for 15 min each at 30% VO_2_	4 weeks	None	
Giles et al. [29] Giles et al. [30] Giles et al. [31] Giles et al. [32]	Crossover	DE 300 three times FA three times	30	30 min of either high intensity cycling (60% VO_2_ peak), low intensity cycling (30% VO_2_ peak), or rest	1 week	None	
Curran et al. [33]	Crossover	DE 300 FA	120	Alternating 20 min of cycling on stationary bike (ventilation 15 L/min/m^2^ BSA) and 40 min of rest	4 weeks	None	
Mookherjee et al. [34]	Crossover	DE 300 FA	120	n/a	4 weeks	Allergen	
Clifford et al. [35]	Crossover	DE 300 + allergen FA + allergen	120	Resting	4 weeks	Allergen	
Kramer et al. [36] Biagioni et al. [37] Carlsten et al. [13] Hosseini et al. [38]	Crossover	DE 300 + allergen FA + allergen	120	Alternating 15 min cycling (ventilation 15 L/min/m^2^ BSA) and 45 min rest	4 weeks	Allergen	
Cliff et al. [39]	Crossover	DE 300 FA	120	2 bouts of 15 min light cycling (ventilation 15 L/min/m^2^)	4 weeks	None	
Rider et al. [40]	Crossover	DE 300 + allergen FA + allergen	120	n/a	4 weeks	Allergen	
Zhang et al. [41]	Crossover	DE 300 + allergen FA + allergen	120	n/a	4 weeks	Allergen	
Carlsten et al. [42]	Crossover	DE 300 + antioxidant DE 300 + placebo FA + placebo	120	Cycling for 15 min every hour at 15 L/min/m^2^ BSA ventilation, otherwise at rest	At least 2 weeks	N-acetylcysteine pre-treatment	
Jiang et al. [43]	Crossover	DE 300 FA	120	Alternating light exercise (15 min) and rest (45 min) on bike (ventilation 15 L/min/m^2^ BSA)	At least 2 weeks	None	
Yamamoto et al. [44]	Crossover	DE 300 + antioxidant DE 300 + placebo FA + placebo	120	15 min cycling per hour (ventilation 15 L/min/m^2^ BSA), otherwise rest	At least 2 weeks	N-acetylcysteine pre-treatment	
Giles et al. [45]	Crossover	DE 300 FA	60	Resting	At least 1 week	None	
Mills et al. [46]	Crossover	DE 300 PDDE Carbon nanoparticles FA	120	Alternating 15 min cycling (ventilation 25 L/min/m^2^ BSA) and 15 min rest	At least 2 weeks	Carbon nanoparticles	Edinburgh University, Edinburgh, UK
Langrish et al. [47]^d^	Crossover	Mills et al. [46]: DE 300 PDDE Carbon nanoparticles FA Barath et al. [86]: DE 250 FA Cruts et al. [91], Mills et al. [93, 96],: DE 300 FA	Mills et al. [46]: 120 Barath et al. [86], Cruts et al. [91], Mills et al. [93, 96]: 60	Mills et al. [46, 96]: alternating 15 min cycling (ventilation 25 L/min/m^2^ BSA) and 15 min rest Barath et al. [86]: alternating 15 min cycling (ventilation 20 L/min/m^2^ BSA) and 15 min rest Cruts et al. [91]: resting Mills et al. [93]: alternating 15 min cycling (ventilation 15 L/min/m^2^ BSA) and 15 min rest	Mills et al. [46, 93]: at least 2 weeks Barath et al. [86]: 22–62 days Cruts et al. [91]: 2–4 days Mills et al. [96]: 2 weeks	Mills et al. [46]: carbon nanoparticles Barath et al. [86], Cruts et al. [91], Mills et al. [93, 96]: none	Mills et al. [46]: Edinburgh University, Edinburgh, UK Barath et al. [86], Cruts et al. [91], Mills et al. [93, 96]: Umea University, Umea, Sweden
Hussain et al. [48]	Crossover	DE 300 FA	60	Alternating 15 min cycling (ventilation 20 L/min/m^2^ BSA) and 15 min rest	1–3 weeks	None	Environmental and Occupational Health Sciences Institute, New Jersey, USA
Pettit et al. [49]	Crossover	DE 300 FA	60	Resting	At least 1 week	None	
Kipen et al. [50]	Crossover	n = 26: DE 200 Secondary organic aerosol FA n = 12: DE 200 FA	120	Resting	At least 1 week	Secondary organic aerosol	
Laumbach et al. [51]	Crossover	DE 300 FA	60	n/a	At least 1 week	Half of the participants had a stressor task (4 min public speaking or 4 min arthimetic problems)	
Huyck et al. [52] Laumbach et al. [53]	Crossover	DE 300 FA	60	Resting	At least 1 week	None	
Pawlak et al. [54]	Parallel	n = 11: DE 100 n = 11: FA	120	n/a	n/a	Live attenuated influenza virus	Environmental Protection Agency Human Studies Facility, Chapel Hill, USA
Stiegel et al. [55] Madden et al. [56]	Crossover	DE 300 DE 300 + O_3_ FA O_3_ For each arm, participants underwent a session with the listed condition *followed by a session of O*_*3*_* next day*	120	Alternating 15 min cycling (ventilation 25 L/min/m^2^ BSA) and 15 min rest	At least 13 days	O_3_	
Tong et al. [57]	Single sequence	DE 100 DE 200 DE 300	120	Resting	At least 2 weeks	None	
Channell et al. [58] Lund et al. [59] Lund et al. [60]	Crossover	DE 100 FA	120	Alternating 15 min cycling (ventilation 25 L/min/m^2^ BSA) and 15 min rest	At least 4 weeks	None	
Noah et al. [61]	Parallel	n = 9: allergic rhinitics exposed to DE 100 n = 7: allergic rhinitics exposed to FA n = 8: healthy subjects exposed to DE 100 n = 8: healthy subjects exposed to FA	120	Resting	n/a	Live attenuated influenza virus	
Pleil et al. [62] Hubbard et al. [63] Sawyer et al. [64] Sobus et al. [65]	Crossover	DE 100 FA	120	Intermittent cycling at 20 L/min/m^2^	3 weeks–6 months	None	
Wauters et al. [66]	Crossover	n = 14: DE 300 + resting AA + resting n = 11: DE 300 + resting AA + resting DE 300 + exercise AA + exercise	DE + resting and AA + resting: 120 DE + exercise and AA + exercise: 60	n = 14: resting n = 11: resting or exercise (alternating 20 min exercise, 20 min rest, 20 min exercise)	At least 1 week	None	Erasme hospital, Bruxelles, Belgium
Wauters et al. [67]	Crossover	n = 10: DE 300 AA n = 8: DE 300 + exercise in hypoxia AA + exercise in normoxia AA + exercise in hypoxia	120	n = 8: exercise in normoxia exercise in hypoxia	At least 1 week	n = 10: dobutamine stress n = 8: exercise in hypoxia	
Wauters et al. [68]	Crossover	n = 7: DE 300 + resting AA + resting n = 5: DE 300 + resting AA + resting DE 300 + exercise	DE + resting and AA + resting: 120 DE + exercise: 60	n = 7: resting n = 5: resting or exercise (two 20 min bouts of exercise at ventilation 20 L/min/m^2^)	At least 1 week	None	
Nightingale et al. [69]	Crossover	DEP 200 FA	120	Resting	4 weeks	None	Imperial College School of Medicine, London, UK
Sawant et al. [70]	Crossover	DE 100 FA FA + NO_2_	120	Four 15 min bouts of moderate cycling on a stationary bicycle	At least 4 weeks	NO_2_	Los Amigos Research and Education Institute (LAREI), Downey, USA
Lu et al. [71]^e^	Crossover	Lund study (Wierzbicka et al. [73]): DE 300 + 46 dB noise DE 300 + 75 dB noise FA + 46 dB noise FA + 75 dB noise EPA study (Pleil et al. [62], Hubbard et al. [63], Sawyer et al. [64], Sobus et al. [65]): DE 100 FA	Lund study: 180 EPA study: 120	Lund study: resting EPA study: intermittent cycling at 20 L/min/m^2^	Lund study: at least 1 week EPA study: at least 3 weeks	Lund study: 46 dB or 75 dB traffic noise EPA study: none	Lund University (Lund, Sweden) and Environmental Protection Agency Human Studies Facility (Chapel Hill, USA)
Hemmingsen et al. [72] Wierzbicka et al. [73] Xu et al. [74]	Crossover	DE 300 + 46 dB noise DE 300 + 75 dB noise FA + 46 dB noise FA + 75 dB noise	180	Resting	At least 1 week	46 dB or 75 dB traffic noise	Lund University, Lund, Sweden
Lucking et al. [75]	Crossover	Protocol 1: DE 350 FA Protocol 2: DE 350 FA	protocol 1: 120 protocol 2: 60	Both protocol 1 and 2: alternating 15 min cycling (ventilation 25 L/min/m^2^ BSA) and 15 min rest	At least 1 week	None	Protocol 1: Edinburgh, UK, protocol 2: Umea University (Umea, Sweden)
Gouveia-Figueira et al. [76] Gouveia-Figueira et al. [77]	Crossover	DE 150 FA	60	Alternating 15 min cycling (ventilation 20 L/min/m^2^ BSA) and 15 min rest	At least 3 weeks	None	Umea University, Umea, Sweden
Behndig et al. [78]^f^	Crossover	Behndig et al. [83] and Larsson et al. [82]: DE 100 FA	120	Alternating 15 min cycling (ventilation 20 L/min/m^2^ BSA) and 15 min rest	At least 3 weeks	None	
Muala et al. [79]	Crossover	DE 350 PDDE (filter A) PDDE (filter B) FA	60	Alternating 15 min cycling (ventilation 20 L/min/m^2^ BSA) and 15 min rest	At least 1 week	None	
Barath et al. [80]	Crossover	DE 300 FA	60	Alternating 15 min cycling (ventilation 20 L/min/m^2^ BSA) and 15 min rest	At least 2 weeks	None	
Langrish et al. [81]	Crossover	DE 300 FA	n/a	Alternating 15 min cycling (ventilation 20 L/min/m^2^ BSA) and 15 min rest	At least 1 week	Study 1 (n = 16): NO synthase inhibitor + sodium nitroprusside + acetylcholine Study 2 (n = 14): NO synthase inhibitor	
Larsson et al. [82]	Crossover	DE 100 FA	120	Alternating 15 min cycling (ventilation 20 L/min/m^2^ BSA) and 15 min rest	At least 3 weeks	None	
Löndahl et al. [22]	Crossover	DE 50 (idling) DE 300 (transient driving)	33	Resting	Different days	None	
Rissler et al. [23]	Crossover	DE 50 (idling) DE 300 (transient driving)	33	Resting	Different days	None	
Behndig et al. [83]	Crossover	DE 100 FA	120	Alternating 15 min cycling (ventilation 20 L/min/m^2^ BSA) and 15 min rest	At least 3 weeks	None	
Lucking et al. [84]	Crossover	DE 300 PDDE FA	60	Alternating 15 min cycling (ventilation 25 L/min/m^2^ BSA) and 15 min rest	At least 1 week	None	
Mills et al. [85]	Crossover	DE 300 FA	60	Alternating 15 min cycling (ventilation 25 L/min/m^2^ BSA for healthy, 15 L/min/m^2^ for CAD subjects) and 15 min rest	At least 2 weeks	None	
Barath et al. [86]	Crossover	DE 250 FA	60	Alternating 15 min cycling (ventilation 20 L/min/m^2^ BSA) and 15 min rest	22–62 days	None	
Sehlstedt et al. [87]	Crossover	DE 300 FA	60	Alternating 15 min cycling (ventilation 20 L/min/m^2^ BSA) and 15 min rest	At least 3 weeks	None	
Langrish et al. [88]	Crossover	DE 300 FA	60	Alternating 15 min cycling (ventilation 25 L/min/m^2^ BSA) and 15 min rest	At least 1 week	None	
Lundbäck et al. [89]	Crossover	DE 350 FA	60	Alternating 15 min cycling (ventilation 25 L/min/m^2^ BSA) and 15 min rest	At least 1 week	None	
Bosson et al. [90]	Crossover	DE 300 + O_3_ FA + O_3_	DE and FA: 60 O_3_: 120	Alternating 15 min cycling (ventilation 20 L/min/m^2^ BSA) and 15 min rest	3–5 weeks	O_3_	
Cruts et al. [91]	Crossover	DE 300 FA	60	Resting	2–4 days	None	
Bosson et al. [92]	Crossover	DE 300 + O_3_ DE 300 + FA	DE and FA: 60 O_3_: 120	Alternating 15 min cycling (ventilation 20 L/min/m^2^ BSA) and 15 min rest	3–6 weeks	O_3_	
Mills et al. [93]	Crossover	DE 300 FA	60	Alternating 15 min cycling (ventilation 15 L/min/m^2^ BSA) and 15 min rest	At least 2 weeks	None	
Törnqvist et al. [94]	Crossover	DE 300 FA	60	Alternating 15 min cycling and 15 min rest	At least 2 weeks	None	
Behndig et al. [95]	Crossover	DE 100 FA	120	Alternating 15 min cycling (ventilation 20 L/min/m^2^ BSA) and 15 min rest	At least 3 weeks	None	
Mills et al. [96]	Crossover	DE 300 FA	60	Alternating 15 min cycling (ventilation 25 L/min/m^2^ BSA) and 15 min rest	2 weeks	None	
Pourazar et al. [97] Pourazar et al. [98] Salvi et al. [99] Salvi et al. [100]	Crossover	DE 300 FA	60	Alternating 15 min cycling (ventilation 20 L/min/m^2^ BSA) and 15 min rest	At least 3 weeks	None	
Mudway et al. [101]	Crossover	DE 100 FA	120	Alternating 15 min cycling (ventilation 15–20 L/min/m^2^ BSA) and 15 min rest	At least 3 weeks	None	
Stenfors et al. [102]	Crossover	DE 100 FA	120	Alternating 15 min cycling (ventilation 15–20 L/min/m^2^ BSA) and 15 min rest	At least 3 weeks	None	
Nordenhäll et al. [14]	Crossover	DE 300 FA	60	Alternating 15 min cycling (ventilation 20 L/min/m^2^ BSA) and 15 min rest	At least 3 weeks	None	
Nordenhäll et al. [103]	Crossover	DE 300 FA	60	Alternating 15 min cycling (ventilation 20 L/min/m^2^ BSA) and 15 min rest	At least 2 weeks	None	
Rudell et al. [104]	Crossover	DE 300 PDDE (filter A) PDDE (filter B) PDDE (filter C) PDDE (filter D) FA	60	Resting	6 days	None	
Rudell et al. [105]	Crossover	DE ? PDDE AA	60	Alternating 10 min cycling on bike at 75 W (15 L/min/m^2^ BSA) and 10 min rest	3 weeks	None	
Blomberg et al. [106]	Crossover	DE 300 AA	60	Alternating 15 min cycling (ventilation 20 L/min/m^2^ BSA) and 15 min rest	At least 3 weeks	None	
Rudell et al. [107]	Crossover	DE ? PDDE AA	60	Alternating 10 min cycling on bike at 75 W and 10 min rest	n/a	None	
Rudell et al. [108]	Single arm	DE?	60	Alternating 10 min cycling on bike at 75 W and 10 min rest	n/a	None	
Tousoulis et al. [109]	Crossover	DE 25 FA	120	n/a	4 weeks	None	University of Athens, Athens, Greece
Vieira et al. [110] Vieira et al. [111]	Crossover	DE 300 PDDE FA	21	15 min rest then 6 min walking without inclination, self-paced	At least 48 h	None	University of Sao Paulo Medical School, Sao Paulo, Brazil
Cosselman et al. [112]	Crossover	DE 200 + placebo FA + placebo DE 200 + antioxidant FA + antioxidant	120	n/a	At least 2 weeks	Ascorbate and N-acetylcysteine pre-treatment	University of Washington, Seattle, USA
Sack et al. [16]	Crossover	DE 200 + antioxidant DE 200 + placebo FA + antioxidant FA + placebo	120	n/a	At least 3 weeks	Ascorbate and N-acetylcysteine pre-treatment	
Carlsten et al. [113]	Crossover	Experiments 1 and 2: DE 100 DE 200 FA Experiment 3: DE 200 + antioxidant DE 200 + placebo FA + antioxidant FA + placebo	120	Resting	2–4 weeks	Experiments 1 and 2: none Experiment 3: antioxidant	
Krishnan et al. [114]	Crossover	DE 200 FA	120	Resting	2 weeks	None	
Cosselman et al. [115]	Crossover	DE 200 FA	120	Resting	At least 2 weeks	None	
Allen et al. [116]	Crossover	DE 200 FA	120	n/a	n/a	None	
Carlsten et al. [117]	Crossover	DE 100 DE 200 FA	120	Resting	At least 2 weeks	None	
Peretz et al. [118]	Crossover	DE 100 DE 200 FA	120	Resting	At least 2 weeks	None	
Peretz et al. [119]	Crossover	DE 100 DE 200 FA	120	Resting	At least 2 weeks	None	
Carlsten et al. [120]	Crossover	DE 100 DE 200 FA	120	Resting	Exposures on 3 different days	None	
Peretz et al. [121]	Crossover	DE 50 DE 100 DE 200 FA	120	Resting	At least 2 weeks	None	

*AA* ambient air, *BSA* body surface area, *CAD* coronary artery disease, *DE* diesel exhaust, *DE 50* diesel exhaust at a target (or achieved concentration, if target concentration was not specified) concentration of 50 μg/m^3^and so on, *DEP* diesel exhaust particles, *DEP 200* diesel exhaust particles at a target concentration of 200 μg/m^3^, *FA* filtered air, *FiO*_*2*_ fraction of inspired oxygen, *min* minute, *NO* nitric oxide, *NO*_*2*_ nitrogen dioxide, *O*_*2*_ oxygen, *O*_*3*_ ozone, *PDDE* particle depleted diesel exhaust, *VO*_*2*_ maximal oxygen uptake

^a^Publications listed in order of research group alphabetically, then by most recent year of publication, then alphabetically by author name. Publications that used the same cohort of participants (or subset of participants from the same cohort) and same exposure arms have been grouped together

^b^Unless otherwise specified (e.g., parallel design), participants were exposed once to each exposure arm listed

^c^Li et al. [25] is derived from the same study as Ryu et al. [15] and Wooding et al. [26], but only uses a subset of the study arms

^d^Langrish et al. [47] uses data pooled from multiple publications, including Barath et al. [86], Cruts et al. [91], Mills et al. [46, 93, 96]. Only data pertaining to DE exposures were considered in this review

^e^Specimens used in Lu et al. [71] were derived from participants in Pleil et al. [62] (EPA study), Hubbard et al. [63] (EPA study), Sawyer et al. [64] (EPA study), Sobus et al. [65] (EPA study), and Wierzbicka et al. [73] (Lund study)

^f^Behndig et al. [78] uses archived biopsies from Behndig et al. [83] and Larsson et al. [82]

During exposure sessions, participants were either at rest or performing exercise on stationary bikes in order to simulate activity levels common to real-world settings and/or increase deposition of inhaled DE. The majority of studies had participants alternate between exercise and rest (only 23 studies had participants rest throughout). Studies that included a cycling component typically standardized exercise intensity by setting a ventilation target, ranging from 15 to 25 L/min/m^2^ body surface area. 11 studies did not report activity of participants during exposure sessions.

Temperature and humidity were maintained at levels generally considered comfortable, between 18 and 26 °C and 35% to 60% RH respectively (Additional file 1). No temperature data was reported in 24 studies, and no humidity data was reported for 30 studies. As mentioned previously, 11 studies referenced a DE generation system (previously detailed) but did not explicitly cite a source for temperature or humidity data—in such cases, temperature and humidity data was assumed also to reflect that noted within the previously article detailing DE generation.

## Summary of particulate matter characteristics across studies

Important components of PM include PM mass concentration, average particle count, and particle size. PM mass concentration is expressed in various size classifications such as coarse particles (PM with aerodynamic diameter under 10 μm but larger than 2.5 μm) and fine particles (PM with aerodynamic diameter under 2.5 μm (PM_2.5_)), etc. [122]. Often for CHE PM mass concentration is used to set the standard at which the DE exposure level is targeted.

**Fig. 1:**
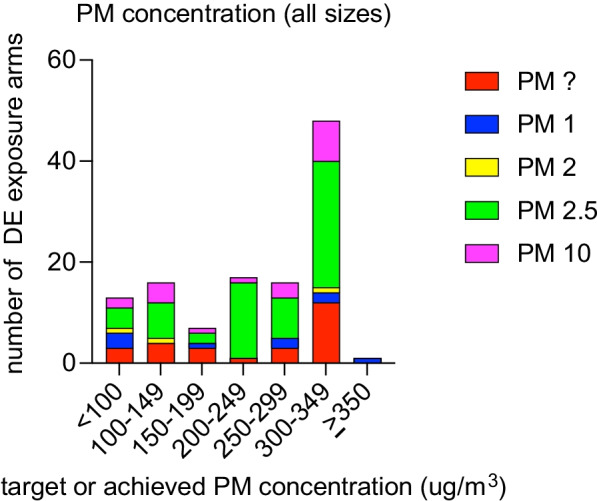
Target or achieved PM concentration for diesel exhaust (DE) exposures within reviewed studies. n = 76 studies, n = 118 DE arms. PM concentration not available for 3 studies (representing 5 arms). Achieved PM concentrations were used when available. Otherwise, target PM concentration was used. When studies included exposures to multiple different PM concentrations (“arms”), each exposure condition contributed to the number of exposure arms. For studies with multiple DE exposure sessions per participant, each session was counted as a separate arm. For studies involving multiple cohorts, exposure conditions for each cohort were counted as separate arms. FA arms not included. See Additional file 1 for full dataset

**Fig. 2:**
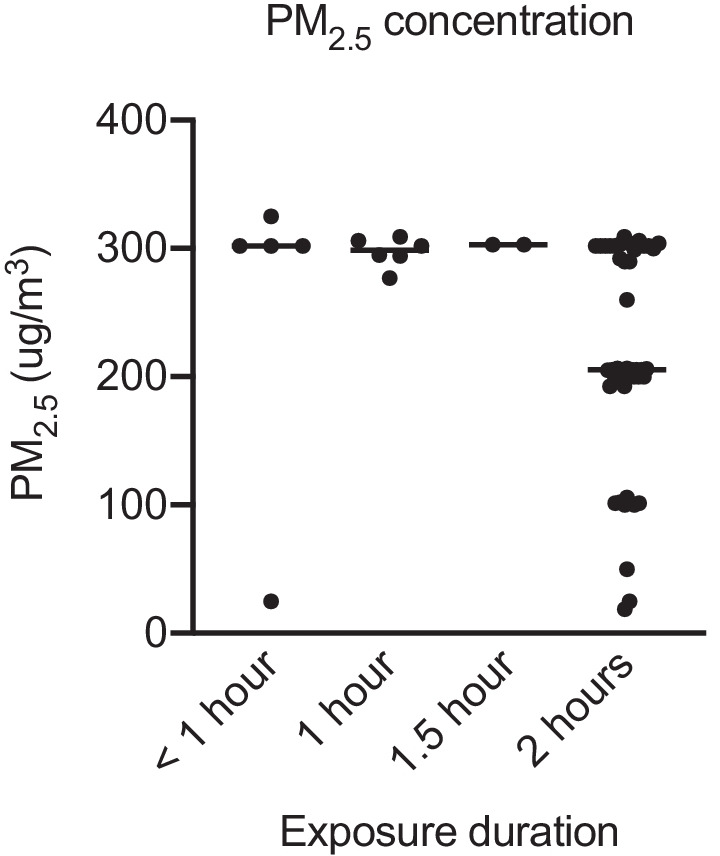
PM_2.5_ concentration for diesel exhaust (DE) exposures of reviewed studies, categorized by duration of exposure. Achieved PM_2.5_ concentration was used if available, otherwise target PM_2.5_ concentration was used. Only studies that reported PM_2.5_ concentrations were included (n = 37 studies). When studies included exposures to multiple different PM concentrations, each exposure condition contributed to the number of exposure arms. For studies with multiple DE exposure sessions per participant, each session was counted as a separate arm. For studies involving multiple cohorts, exposure conditions for each cohort were counted as separate arms. This resulted in a total of n = 59 exposure arms. FA arms not included. See Additional file 1 for full dataset

Of the studies reviewed, the majority (37) reported and targeted PM mass concentration levels as PM_2.5_. 19 studies reported PM_10_ concentration (PM with aerodynamic diameter under 10 μm), and the remaining reported PM_1_ (PM with aerodynamic diameter under 1 μm), PM_2_ (PM with aerodynamic diameter under 2 μm), or PM without specifying size. PM concentration was not available for 3 studies (Additional file 1). The most common target mass concentration for the DE exposures reviewed was a PM_2.5_ concentration of 300 μg/m^3^ (Fig. 1), roughly one order of magnitude above the PM_2.5_ 24-h standards set by the US EPA [123] and World Health Organization [124]. For DE exposures, the highest PM_2.5_ concentration used was 325 μg/m^3^ and the lowest PM_2.5_ concentration used was 19 μg/m^3^ (Fig. 2). 8 studies involved a particle depleted DE (PDDE) exposure arm—the PM concentration of these arms were mostly under 100 μg/m^3^ (Additional file 1). 1 study exposed participants to resuspended DEP [69]. For FA exposures, the highest PM_2.5_ concentration was 21 μg/m^3^ and most studies used a PM_2.5_ concentration under 10 μg/m^3^ (Additional file 1).

46 studies reported average particle count for the DE exposure component while only 28 studies reported particle counts for FA exposures (Additional file 1). The particle count range for reviewed DE exposures were 30 particles/cm^3^—5.4 × 10^6^ particles/cm^3^ and 14 particles/cm^3^—1.7 × 10^4^ particles/cm^3^ for FA exposures (Additional file 1). There were 8 studies that included PDDE exposures (Additional file 1) and the particle counts for these studies were similar to those of FA exposures. Methodology for reported particle count in Blomberg et al. [106] was unclear and therefore not included in analysis.

Particle size was also often reported, with 39 studies reporting particle size for DE exposures and 13 for FA exposures. A variety of methods and aerosol size measurements were reported, including most commonly mass median diameter, count median diameter and geometric mean (see Additional file 1). It should be noted that for both particle count and particle size measurements that different particles size range distributions were use which may affect inter-study comparisons.

Detailed particle composition was rarely reported, with only 17 publications reporting carbon composition and 4 reporting particle polyaromatic hydrocarbon (PAH) concentration. No publications reviewed reported metallic composition of DE, though on occasion the studies referenced earlier work that did so from the same lab [125].

## Gaseous components of exposures

**Fig. 3:**
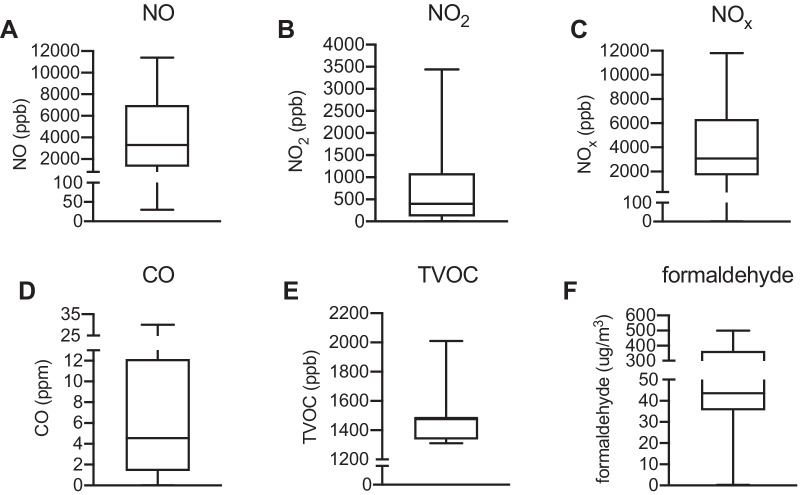
Gaseous portion of diesel exhaust (DE) exposures in reviewed studies. Whiskers denote minimum and maximum values, box denotes 25th and 75th percentiles, line denotes median. When studies included exposures to multiple different PM concentrations, each exposure condition contributed to the number of exposure arms. For studies with multiple DE exposure sessions per participant, each session was counted as a separate arm. For studies involving multiple cohorts, exposure conditions for each cohort were counted as separate arms. **A** nitric oxide (NO), n = 63 studies, n = 95 arms; **B** nitrogen dioxide (NO_2_), n = 69 studies, n = 107 arms; **C** nitrogen oxides (NO_x_), n = 41 studies, n = 61 arms; **D** carbon monoxide (CO), n = 62 studies, n = 93 arms; **E** total volatile organic compounds (TVOC), n = 13 studies, n = 19 arms; **F** formaldehyde, n = 14 studies, n = 21 arms. For Noah et al. [61] and Pawlak et al. [54], concentrations of the gaseous portion of exposures were listed as ranges. As such, the median of each range was used in this figure. See Additional file 1 for full dataset

Commonly reported gaseous components of DE exposures included NO, NO_2_, NO_x_, CO, TVOC, and formaldehyde (Additional file 1). There was no data available for the gaseous portion of DE exposures for 4 studies. Of the gaseous components that were characterized, each showed a wide range in concentration (Fig. 3). In general, concentrations in DE exposures were greater than air quality standards set by the US National Ambient Air Quality Standard (NAAQS), although those metrics are calculated differently. For example, over 75% of studies had concentrations of NO_2_ greater than the 1-h US NAAQS standard of 100 ppb, but the latter is 3-year average of 98th percentile of the yearly distribution of 1-h daily maximum concentrations within which significantly higher levels such as those in CHEs intermittently occur [123]. Just under half of studies exceeded the NAAQS 1-h average of 35 ppm for CO [123].

Gaseous pollutant composition of FA exposures was not available for 40 publications (4 studies did not have a FA exposure condition) (Additional file 1). If FA composition data was included, often fewer parameters were reported compared to the DE condition. Though FA exposures are used as a control condition, completeness of reporting can assure readers of the validity of any conclusions made.

## Study participant characteristics

The average sample size reported was 22 participants. 11 studies involved ≤ 10 participants, 43 studies involved 11–20 participants, 11 studies involved 21–30 participants, with the remaining involving more than 30 participants (Additional file 1). The largest sample size reported was 97 participants [51]. Participants were typically aged between 20 and 40 (Additional file 1), with the full range between 18 and 80 [28]. 11 studies reported participants over 50 years old (Additional file 1). Some studies reported only a mean and standard variation with respect to age, rather than an age range or a complete list of participant ages.

**Fig. 4:**
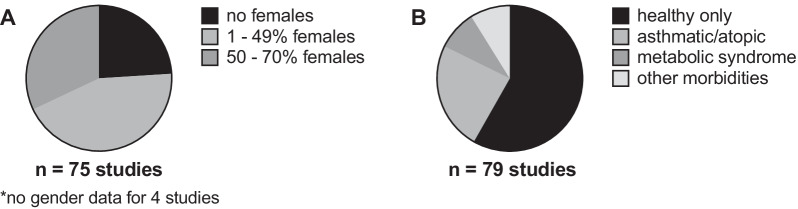
Participant sex* and underlying phenotype. **A** Studies by percentage of female participants. **B** Studies by participant type. Studies that included healthy, non-asthmatic, non-atopic participants only were categorized under healthy. Studies involving healthy participants that were not screened for atopy were categorized under healthy. Studies that included at least some participants with positive skin prick testing, positive methacholine challenge (typically PC_20_ ≤ 8 mg/mL), diagnosed asthma, exercise-induced bronchoconstriction, or other atopic diseases (even if healthy participants, with none of these conditions, were also included) were categorized under “asthmatic/atopic”. Metabolic syndrome for most studies was classified according to criteria outlined in [126]. See Additional file 1 for full dataset. *Most studies categorized participants by biological sex though some reported participant gender (self-identification as female or male). As the potential difference between sex and gender was not carefully elaborated in these studies, the term “sex” is used herein (recognizing that in some cases sex and gender may not correspond but that we do not have the data resolution to address this further)

There was a male predominance in participant sex (Fig. 4). A fifth of reviewed studies only included male participants, and roughly two-thirds of studies included less than 50% female participants (Fig. 4). Pregnancy is one of the most common reasons females are excluded from biomedical experiments, and several of the reviewed studies screened for pregnancy prior to participation. Some studies also considered hormonal variations related to the menstrual cycle confounding, and either timed exposures to the first half of the menstrual cycle [16, 115] or excluded females altogether [68, 86].

Common participant exclusion criteria for CHE were medical comorbidities, regular medication use (including vitamin supplements or antioxidants), current smoking, and significant occupational exposure to air pollution (Additional file 1). Only a single study included current smokers [109]. Similarly, while most studies listed significant occupational exposure to air pollution in their exclusion criteria, one publication included a cohort of bus drivers that were often exposed to DE [104].

58% of studies included healthy participants only (Fig. 4). Healthy participants were typically defined as those without cardiovascular disease, respiratory disease, or other chronic medical conditions. Some publications conducted physical exams, electrocardiography, or spirometry to screen participants. 24% of studies included asthmatic and/or atopic individuals, characterized by either positive skin prick testing, positive methacholine challenge, physician diagnosed asthma, or other diagnosis such as allergic rhinitis (Fig. 4). Most studies defined positive methacholine challenge defined as a provocative concentration of methacholine resulting in 20% decrease in FEV_1_ (PC_20_) less than or equal to 8 mg/mL, though some used cut offs of less than 8 mg/mL or less than 16 mg/mL. The majority of studies that included asthmatic or atopic participants performed spirometry and methacholine challenge testing as screening measures, likely due to the high prevalence of asthma misdiagnosis [127–129].

9% of studies included participants with metabolic syndrome, most often defined according to the American Heart Association and National Heart, Lung, and Blood Institute criteria [126]. This population is of special interest as individuals are at higher risk of cardiovascular disease [130] and DE exposure is known to promote vascular dysfunction and thrombosis [75, 81, 96, 119]. Metabolic syndrome is also associated with chronic oxidative stress [131], one of the likely mechanisms for DE-mediated effects [112].

Only a small number of studies included participants with other significant chronic medical conditions, such as COPD [22, 28], coronary heart disease [47, 85, 93], and heart failure [110, 111]. These populations were thought potentially more susceptible to adverse events from DE exposure, such that their inclusion in CHE experiments has been limited.

## Discussion

### Diesel characterization and study design recommendations

As the literature of CHE–DE studies expands, a standard of DE characterization and data reporting should be considered. This standardization will facilitate not only a level of quality assurance in detailed reporting but also allow for ease of inter-publication comparison of results. We now outline some recommendations that should lead to a more complete exposure profile and we also highlight how these parameters could influence reported health outcomes.

The characteristics of DE can greatly affect health outcomes. Therefore, much care should be taken to provide a complete, accurate and detailed profile of exposure levels. PM_2.5_ is commonly regarded as the PM fraction most damaging to human health [122] as these particles are significantly deposited within the respiratory tract, with smaller particles generally penetrating deeper [132]. A study conducted in the US reported that a 10 μg/m^3^ increase in PM_2.5_ increased cardiovascular mortality risk by 8–18% [133]. PM_2.5_ exposure has been shown to be a stronger predictor of increased mortality risk than PM_10_ exposure [134, 135], suggesting that the coarse fraction, while not benign, may be relatively less hazardous. Given the influence of PM size on health outcomes, standardizing the concentration (or range of concentrations) and also the PM size fraction used to determine this concentration, and clearly reporting as such would allow for easier comparisons between studies. Furthermore, reporting particle number (ideally, using a standardized definition), would be helpful for interpretation and comparison to the epidemiologic and toxicologic literature. With respect to determining an optimal PM concentration for study, we discuss the challenges associated with such a task in a separate companion paper currently under review.

Gaseous composition of DE was often not remeasured for each study and, instead, data from older studies conducted by the same lab were cited instead. However, the composition of DE produced by the same DE generation system depends on a multitude of factors that can be difficult to control over time. The same engine will accumulate wear and tear with use, fuel can vary in spite of attempts to keep uniform, and the time from last maintenance (oil change, etc.) can all influence the resultant DE generated. For example, three separate studies from the Air Pollution Exposure Laboratory in Vancouver, Canada [125] reported different concentrations of some aerosol components despite using the same engine, type of fuel, engine load, dilution system, and target PM_2.5_ concentration [13, 32, 36]. Though pollutant composition can be difficult to maintain precisely, standard parameters to be reported for each separate experiment can at least aid in interpretation of results.

Activity level during exposure should be carefully considered in design and reported with study results, as it can influence outcomes. One study compared platelet activation in participants exposed to DE while rest or alternating between exercise and rest [66]. Exercise increased particulate inhalation, and platelet activation was significantly increased in the exercise group compared to the resting group [66]. Exercise-induced increases in ventilation likely enhances inhalation of not just particles, but also of gaseous components of air pollution as well. As such, further design for, and detailing of, DE exposure at different levels of activity is likely to reveal helpful data.

As with all biological investigations, sex- and/or gender-specific differences are important to uncover and greater efforts should be made to include female participants in CHE experiments. Studies done in mouse models have illustrated sex-dependent effects of DE exposure. Intranasal inoculation of DE particles induced a greater degree of pulmonary neutrophilia and impairment of lung function in female mice compared to male mice [136]. In a different study, inhalation of DE was associated with increased inflammatory markers in mouse brain, an effect that was more marked in males [137]. CHE–DE studies have typically not found differential health effects based on sex. Furthermore, and most importantly, CHE–DE studies to date have not generally done careful analysis of this, if at all, and when examined may not have done so in a sex-disaggregated fashion, as now recommended. Furthermore, the male predominance of participants in these studies may have disallowed revelation of any potential differences. Investigation of effect modification by sex and gender, as well as sex- and gender-specific (disaggregated) responses, in humans exposed to DE is a compelling future direction for CHE studies.

Finally, it was often the case that results from one study were reported across multiple publications, though it was not always clear when this was the case. This review attempted to cluster publications using the same participants and exposure sessions based on in-text references as well as clinical trial number, however references to other publications utilizing the same or overlapping participant-exposures were sometimes not expressly stated. To facilitate transparency in this regard, future publications should explicitly reference all other publications stemming from the same participant-exposure session cluster, the location and time period over which the study was conducted, as well as clinical trial number.

### Changing diesel engine technology and fuel

The emissions standards of on-road vehicles, diesel engines in particular, are ever-evolving.

The EPA’s most recent set of emission standards for light duty vehicles was phased in, beginning with model year 2017, and will be fully implemented by the year 2025 [138]. These updated standards significantly reduce the allowed emissions of PM and gaseous components, including NO_x_, formaldehyde, and CO in vehicle emissions [138]. The EPA has been also phasing in new standards with respect to fuel consumption and greenhouse gas emissions, beginning in 2014 for medium and heavy-duty vehicles and 2017 for light-duty vehicles [139, 140]. Regulations for vehicle emissions are regularly updated in Europe as well, where the most recent sets of standards, Euro 6 for light-duty vehicles and Euro VI for heavy-duty vehicles, came into effect in 2015 and 2013 respectively [141]. Therefore, published studies will tend to reflect older technology. However, it is important to recognize that the ‘typical’ diesel engine in use worldwide today is not one of the most recent and technologically advanced models. Instead, given the hardy and resilient nature of diesel engines, most engines in use globally at any given moment remain those of years and decades past, such that the studies reviewed herein remain highly relevant (and, arguably, more relevant than are the most recent models given that they remain in the minority overall).

In recent years, CHE–DE studies have also trended towards using low-sulfur diesel fuels, likely reflecting the global trend towards reducing sulfur content in fuels. In the mid-2000’s, the US began restricting diesel sulfur content to under 15 ppm [142] while the EU and Japan set an upper limit of 10 ppm [143]. Sulfur increases the emission of pollutants such as sulfur dioxide (SO_2_), CO, NO_x_, and PM [143]. Given the evolving nature of emissions control regulations and technology, care should be taken to ensure both the fuel and engines used in research are updated in tandem with those used in the real world although, as noted similarly for engines, much of the world lags considerably behind the ‘leading edge’ of such advances.

### Limitations of controlled human exposures

While CHE experiments lend themselves well to investigating acute effects of DE inhalation, the relationship of effects to those of chronic exposure (conceptually a series of such acute effects) is yet uncertain. Long-term DE exposure drives chronic disease development and progression [144–146] and so CHE studies therefore are not ideally suited to shed light on such disease. Although pathophysiology of chronic disease may be understood as resulting from an accumulation of ‘hits’ of recurrent acute exposures, it remains unclear whether it is transient exposure peaks or rather longer-term exposures more modestly above background levels, or perhaps more likely a combination of both, that are most influential in this regard. Furthermore, CHE experiments are of necessity somewhat circumscribed and simplified in their design, and thus cannot capture the full complexity of real-world exposures. Given varying sources of DE, dynamic concurrent exposures, and fluctuating pollutant composition and PM concentration, a plethora of variables underlie the actual settings in which people breathe [147]. Additionally, numbers and phenotypes of participants are limited due to practical considerations, so results being extrapolated to larger and broader populations must be done with caution and circumspection. Where possible, careful inclusion of individuals with chronic conditions in CHE experiments can yield valuable data that will greatly benefit these susceptible populations. Finally, as discussed above, engine technology evolves over time, posing another caveat to interpretation and application of historical results.

## Conclusion

Studies of controlled human exposures (CHE) to diesel exhaust, a paradigm of traffic-related air pollution, are invaluable within the armamentarium of investigations that elucidate effects of (and ways to protect from) the air we breathe. However, there is considerable variability in the study design and reporting of exposure parameters across CHE experiments. Standardization and greater detail in reporting elements such as pollutant composition, PM, and particle diameter will allow stronger comparisons to be drawn. There is a male predominance in CHE studies, and strident efforts should be made to include female participants. Most studies included healthy and relatively young participants only; inclusion of older and more diseased populations has proven safe in carefully designed CHE studies to date and is recommended into the future, to deepen insight regarding the full range of impact of traffic-related air pollution on global populations. No CHE–DE studies to date have been performed with photochemical aging similar to that expected in ambient conditions significantly distant from point sources (and thus reflective of realistic secondary ambient aerosols) and CHE–DE that better recapitulate these conditions are desired, though CHE studies to CAP do account for the effects of aging to an extent. Finally, more and larger CHE studies of interventions to protect from adverse effects should be performed, in parallel to vigorous efforts to forestall exposures at their root.

## Supplementary Information

**Additional file 1**. All study details extracted from reviewed publications are listed in the supplemental material: details of diesel exhaust generation, temperature and humidity of exposure sessions, PM and gaseous pollutant composition of diesel exhaust exposures, PM and gaseous pollutant composition of filtered air exposures, particle diameter and particle count of diesel exhaust exposures and filtered air exposures, and participant demographics. Supplemental material is organized by research group alphabetically, then by most recent year of publication to least recent, then alphabetically by author name. Publications that use the same cohort of participants (or a subset of the same participants) and the same exposure arms have been grouped together. Additional details (abbreviations, etc.) are listed within the supplemental material file.

## Data Availability

All data reviewed or described are included in this published article and its supplementary information file.

## Abbreviations

AA: Ambient air
BSA: Body surface area
CAD: Coronary artery disease
CHE: Controlled human exposure
CHE–DE: Controlled human exposure to diesel exhaust
CO: Carbon monoxide
COPD: Chronic obstructive pulmonary disease
DE: Diesel exhaust
DEP: Diesel exhaust particles
EC: Elemental carbon
EPA: Environmental Protection Agency
EU: European Union
FA: Filtered air
FEV_1_: Forced expiratory volume in the first second
FiO_2_: Fraction of inspired oxygen
HC: Hydrocarbon
NAAQS: National Ambient Air Quality Standard
NO: Nitrogen monoxide
NO_x_: Nitrogen oxides
NO_2_: Nitrogen dioxide
OC: Organic carbon
O_2_: Oxygen
O_3_: Ozone
PAH: Polyaromatic hydrocarbon
PC_20_: Provocative concentration of methacholine resulting in 20% decrease in FEV1
PDDE: Particle depleted diesel exhaust
PM: Particulate matter
SO_2_: Sulfur dioxide
TRAP: Traffic related air pollution
TVOC: Total volatile organic compounds
VO_2_: Maximal oxygen uptake
